# An Overview of Ten Italian Horse Breeds through Mitochondrial DNA

**DOI:** 10.1371/journal.pone.0153004

**Published:** 2016-04-07

**Authors:** Irene Cardinali, Hovirag Lancioni, Andrea Giontella, Marco Rosario Capodiferro, Stefano Capomaccio, Luca Buttazzoni, Giovanni Paolo Biggio, Raffaele Cherchi, Emidio Albertini, Anna Olivieri, Katia Cappelli, Alessandro Achilli, Maurizio Silvestrelli

**Affiliations:** 1 Dipartimento di Chimica, Biologia e Biotecnologie, Università di Perugia, Perugia, Italy; 2 Centro di Studio del Cavallo Sportivo, Dipartimento di Medicina Veterinaria, Università di Perugia, Perugia, Italy; 3 Dipartimento di Biologia e Biotecnologie “L. Spallanzani”, Università di Pavia, Pavia, Italy; 4 Centro di ricerca per la produzione delle carni e il miglioramento genetico, Sede centrale–Monterotondo, Roma, Italy; 5 Agenzia per la ricerca in agricoltura–AGRIS Sardegna, Sassari, Italy; 6 Dipartimento di Scienze Agrarie, Alimentari ed Ambientali, Università di Perugia, Perugia, Italy; University of Florence, ITALY

## Abstract

**Background:**

The climatic and cultural diversity of the Italian Peninsula triggered, over time, the development of a great variety of horse breeds, whose origin and history are still unclear. To clarify this issue, analyses on phenotypic traits and genealogical data were recently coupled with molecular screening.

**Methodology:**

To provide a comprehensive overview of the horse genetic variability in Italy, we produced and phylogenetically analyzed 407 mitochondrial DNA (mtDNA) control-region sequences from ten of the most important Italian riding horse and pony breeds: Bardigiano, Esperia, Giara, Lipizzan, Maremmano, Monterufolino, Murgese, Sarcidano, Sardinian Anglo-Arab, and Tolfetano. A collection of 36 Arabian horses was also evaluated to assess the genetic consequences of their common use for the improvement of some local breeds.

**Conclusions:**

In Italian horses, all previously described domestic mtDNA haplogroups were detected as well as a high haplotype diversity. These findings indicate that the ancestral local mares harbored an extensive genetic diversity. Moreover, the limited haplotype sharing (11%) with the Arabian horse reveals that its impact on the autochthonous mitochondrial gene pools during the final establishment of pure breeds was marginal, if any. The only significant signs of genetic structure and differentiation were detected in the geographically most isolated contexts (i.e. Monterufolino and Sardinian breeds). Such a geographic effect was also confirmed in a wider breed setting, where the Italian pool stands in an intermediate position together with most of the other Mediterranean stocks. However, some notable exceptions and peculiar genetic proximities lend genetic support to historical theories about the origin of specific Italian breeds.

## Introduction

A great variety of horse breeds developed, over time, in various Italian cultural contexts and geographic habitats. Light horses (hotblood/warmblood; withers height: 148–170 cm) are typical of the drier central and southern regions, while the northern wet regions are characterized by heavy horses (coldblood; withers height: 148–165 cm). Harsh conditions of marginal and insular areas fostered the smaller size horses (ponies; withers height: 115–147 cm). Until the 1940s horse breeding was mainly linked to the production of animals for military purposes, agricultural labors, forestry and local carriages. Beginning in the fifties, the mechanization of agriculture and transportation caused a rapid decline of horse breeding; such trend has been currently mitigated by a renewed cultural interest in rural life. Most recently, the increased leisure-time physical activities have resulted in a growing consideration and demand for “riding horses”; riding refers to the use of horses for leisure/pleasure purposes including competition events (jumping, driving, flat racing, etc.). In Italy, the demand for riding horses includes: cosmopolitan breeds (Thoroughbreds and Arabs), many autochthonous Italian breeds described in Studbooks, many local Italian populations with “Anagraphic Register of equine populations identifiable as local ethnic groups” and several crossbreedings between all of them.

Phenotypic traits and genealogical data are often insufficient to ascertain the horse history and origin. Molecular analyses provide a needful and reliable tool that can be employed along with the morphometric approach and traditional breeding strategies for an efficient management of genetic resources [1]. Due to its high mutation rate, lack of recombination and maternal inheritance, the control region of the mitochondrial DNA (mtDNA) is a powerful marker system for phylogenetic and phylogeographic studies. MtDNA studies on horses have proved to be capable to identify intra- and interbreed relationships [2–9], particularly when combined with historical information [2, 10, 11]. Unfortunately, most previous studies have been carried out on a very short and hypervariable segment (~350 bp) of the control region (HVSI: nucleotide positions 15,469–15,834) [10, 12–15]. In 2013 Khanshour and Cothran [9] have shown in Arabian horse populations that the degree of informativeness can be extensively improved by increasing the length of the analyzed mtDNA control-region sequence. Most recently, similar to many other livestock species [16–18] also the sequence variation of the entire equine mitogenome was investigated [19–21], contributing extensively to our current understanding of the domestication process. Seventeen different mtDNA haplogroups were identified in domestic breeds leading to the conclusion that the domestication of the wild horse, *Equus ferus*, has been a widespread process that persisted for several thousands of years (throughout the Neolithic) and occurred at different places, mostly centered in the Western Eurasian steppes [22], as also suggested by archeological evidences [23]; but possibly also in Western Europe [19]. The spread of domestic herds across Eurasia involved an extensive introgression from the wild; in particular, it has been proposed that the horse was introduced in Italy with the arrival of Indo-European populations in the Bronze Age and used for military, riding and agricultural purposes [24].

Despite the pivotal role that horses have played in human society’s development, multiple aspects of modern breeds’ origin and history remain unclear. In Italy, several local breeds have reached a national recognition due to their phenotypic characteristics and to particular socio-cultural and productive peculiarities (a complete list is available at http://www.fao.org/dad-is/). However, genetic studies of Italian horse breeds are still limited [25–28] and there are only a few examples of maternal inheritance investigations, but they generally focused on a specific geographic area [14, 29, 30] or included a limited number of samples per breed [31, 32].

**Fig 1:**
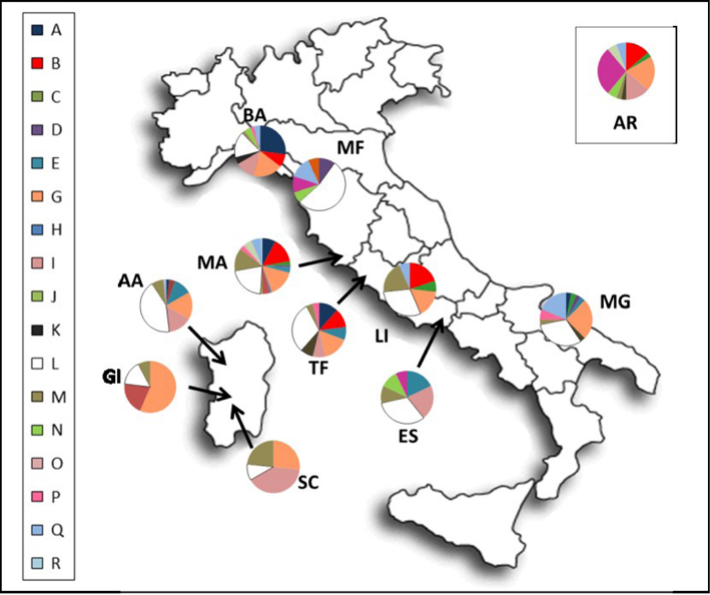
Sampling locations and frequency distributions of mitochondrial haplogroups. Breed code as in Table 2.

To obtain a more comprehensive overview of the Italian horse mitochondrial gene pool we have here determined and phylogenetically analyzed the mtDNA control-region variation of 407 horses from ten of the most important Italian riding horses (including hotblood/warmblood horses and ponies): Bardigiano, Esperia, Giara, Lipizzan, Maremmano, Monterufolino, Murgese, Sarcidano, Sardinian Anglo-Arab and Tolfetano (Fig 1 and Table 1).

**Table 1:** List of Italian breeds analyzed in this paper.

Breed	Current Distribution in Italy	Breeding Conditions	Breed Consistency^a^	Genealogical Records	Withers	Breed Classification (AIA)^b^	Breed Classification (FAO)^c^
**Bardigiano**	North (Emilian Apennine)	Controlled	2606	Studbook	Pony	Autochthonous breed	National breed
**Monterufolino**	Centre (Tuscany)	Semi-feral^d^	252	Anagr. Reg.	Pony	Autochthonous breed	Local breed (Critical)
**Maremmano**	Centre (Tuscany and Latium)	Controlled	6300	Studbook	Horse	Autochthonous breed	National breed
**Tolfetano**	Centre (Latium)	Semi-feral	1518	Anagr. Reg.	Pony	Autochthonous breed	Local breed (Endangered)
**Lipizzan**	Centre (Latium)	Controlled	433	Studbook	Horse		International breed
**Esperia**	Centre (South Latium)	Semi-feral	1373	Anagr. Reg.	Pony	Autochthonous breed	Local breed (Endangered)
**Murgese**	South (Apulia)	Controlled	5564	Studbook	Horse	Autochthonous breed	National breed
**Sardinian Anglo-Arab**	Sardinia	Controlled	9606	Studbook	Horse	Autochthonous breed	National breed
**Giara**	Sardinia	Semi-feral	518	Anagr. Reg.	Pony	Autochthonous breed	Local breed (Endangered)
**Sarcidano**	Sardinia	Semi-feral	110	Anagr. Reg.	Pony	Autochthonous breed	Local breed

^a^ Breed consistency = number of individuals recorded in Studbooks or Anagraphic Registers as in 2015 [33, 34].

^b^ Italian breeders' association. The Ministerial Decree n. 1598 of January 23, 2015 reported fifteen indigenous horse breeds recorded in the Italian Registry of Autochthonous Equine Breeds.

^c^ Based on the establishment of Studbooks or Anagraphic Registers.

^d^ Free-roaming horse breeds of domesticated ancestry requiring minimal human management (e.g. supplemental feeding, vaccinations).

## Results and Discussion

### An overview of the mtDNA sequence variation

More than half of the mtDNA control region, precisely 610 bps (from np 15491 to np 16100), was sequenced in all 407 Italian samples. An additional collection of 36 Arabian horses, which were heavily used in the improvement of some Italian breeds, was analyzed and used as an external reference group. Overall, we identified from seven to 52 haplotypes in the different Italian breeds and 14 in the Arabian horses, summing up to a total of 126 distinct haplotypes. Seventy-eight were unique (found only in a single Italian breed) while 34 were shared among different Italian breeds. Only four haplotypes were in common between Italian and Arabian horses (S1 Table) and these might represent the legacy of recent maternal gene flow from Arabian horses into Italian breeds. Taking into account that the four haplotypes encompass only eleven horses [Maremmano (5), Lipizzan (3) and Sardinian Anglo-Arab (1) horses, Bardigiano (1) and Esperia ponies (1)], this observation indicates that the Arabian horse contributed at most marginally in the formation of the modern mtDNA gene pools of these breeds; this is in agreement with the scenario that the introgression from the Arabian horse was stallion-mediated.

The overall sequence alignment of Italian samples revealed 91 polymorphic sites (S), represented by 90 transitions and three indels (two deletions at nps 15532 and 15868, and one insertion at np 16063; we found also a transition at nps 15868 and 16063) (Table 2).

**Table 2:** Estimates of genetic diversity^a^.

Breed	CODE	N	π	Nh	Hd	S
**Bardigiano**	BA	48	0.0197	24	0.957	59
**Monterufolino**	MF	30	0.0220	7	0.796	49
**Maremmano**	MA	90	0.0206	53	0.980	75
**Tolfetano**	TF	26	0.0202	18	0.966	46
**Lipizzan**	LI	30	0.0214	11	0.916	46
**Esperia**	ES	28	0.0174	8	0.869	37
**Murgese**	MG	32	0.0219	21	0.968	55
**Sardinian Anglo-Arab**	AA	54	0.0192	31	0.970	49
**Giara**	GI	39	0.0166	9	0.888	33
**Sarcidano**	SC	30	0.0174	8	0.839	33
**Italian Breeds**		**407**	**0.0200**	**116**	**0.979**	**91**
**Arabian horse**	AR	36	0.0175	14	0.881	44
**Total**		**443**	**0.0200**	**126**	**0.981**	**93**

^a^ N = number of analyzed samples; π = nucleotide diversity; Nh = number of haplotypes; Hd = haplotype diversity; S = number of polymorphic sites.

Nucleotide diversity (π) across all Italian horses was estimated at 0.020. Haplotype diversity was also very high (Hd = 0.979), confirming what already seen in previous horse mtDNA studies [8, 29, 31, 32, 35]. We detected the highest haplotype diversity in the Maremmano horse (Hd = 0.980), followed by the Sardinian Anglo-Arab (Hd = 0.970). The lowest value (Hd = 0.796) was registered in the Monterufolino breed.

The analysis of molecular variance (AMOVA) established that the majority of the observed variance is attributable to differences among samples within breeds (93.57%). However, the remaining among-breeds’ component of genetic variation (6.43%) could be associated with a significant value of the fixation index (Φ_ST_ = 0.064, *p-value* < 0.001). We examined different possible structures by establishing and comparing different population groups, which were artificially created by considering various features in turn, such as: breeding conditions (semi-feral *vs* controlled); height at the withers (ponies *vs* others); geographic prevalence (e.g. indigenous of Sardinia *vs* others). Actually, the only significant sign of genetic differentiation was found between the two local Sardinian breeds (Giara and Sarcidano) and the other breeds (Table 3), particularly when considering Monterufolino as a third independent group (Φ_CT_ = 0.063, *p-value* < 0.001).

**Table 3:** Hierarchical AMOVA table.

Source of variation	df	Variance component	Variance (%)	Fixation index^a^	P-value^b^
**Between areas (Sardinia vs others)**	1	0.354	5.18	Φ_CT_ = 0.052	0.029*
**Among populations within areas**	8	0.311	4.54	Φ_SC_ = 0.048	0.000***
**Within populations**	397	6.172	90.28	Φ_ST_ = 0.097	0.000***

^a^ Φ_CT_ = variation among groups divided by total variation, Φ_SC_ = variation among sub-groups divided by the sum of variation among sub-groups within groups and variation within sub-groups, Φ_ST_ = the sum of variation groups divided by total variation.

^b^ ns = *P* > 0.05

* = *P* ≤ 0.05

*** = *P* ≤ 0.001.

This is consistent with the genetic distances between populations: Monterufolino is genetically the most distant breed, while Giara and Sarcidano are confirmed as the most closely related (S1 Fig; pairwise distances above diagonal and Nei’s distances below diagonal).

### Phylogenetic analyses and haplogroup classification

**Fig 2:**
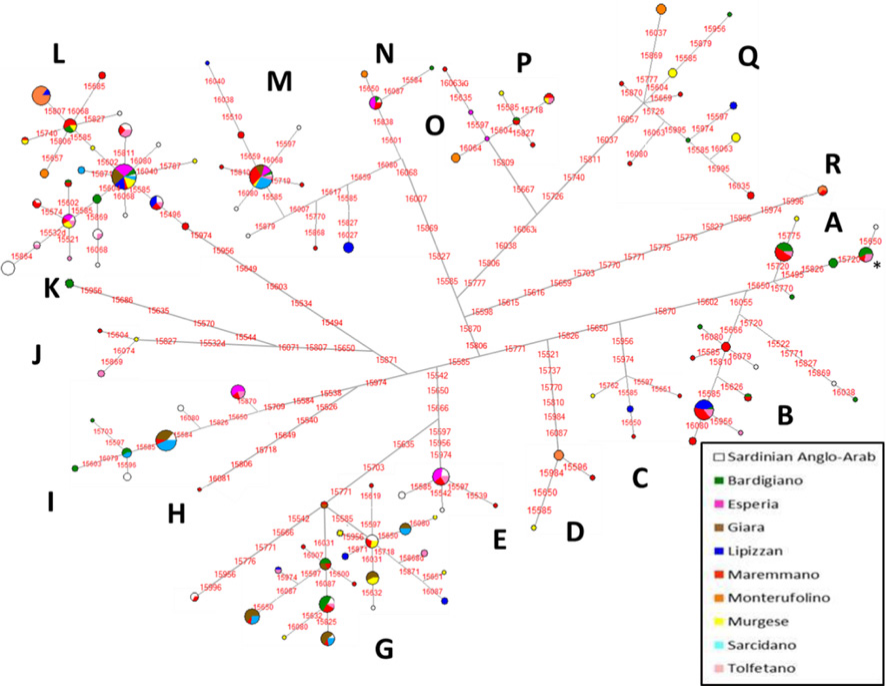
Median-Joining Network based on control-region sequences of ten Italian horse breeds. The asterisk indicates the haplotype identical to ERS.

The reconstructed network of the control-region sequences (Fig 2) clearly defines some major branches corresponding to the horse haplogroups identified so far [19].

The haplogroup classification was confirmed and refined through an accurate analysis of diagnostic mutational motifs identified in the control-region haplotypes (S1 Table). As expected, the Przewalski’s specific haplogroup F was absent in our batch of domestic horses. The stochastic distribution of our haplotypes among the remaining 17 haplogroups confirms that it is not possible to identify breed-specific mitochondrial clades, at least at this level of resolution. About one fourth (N = 109) of the 407 Italian samples carries the haplogroup L mutational motif (nps 15494, 15495 and 15496), which was often reported as the most common in a wide range of Italian (Bardigiano, Giara, Haflinger, Italian Heavy Draught, Italian Trotter, Lipizzan, Maremmano, Murgese, Sanfratellano, Sarcidano, Sicilian Indigenous and Ventasso horse) and Western Eurasian breeds [6, 8, 19, 29–32, 36–38]. Haplogroup L is also the most common in seven Italian breeds analyzed in this work, while it is absent among the Arabian samples (Table 4).

**Table 4:** Haplogroup frequencies (%) in ten Italian breeds and Arabian horses^a^.

Breed	A	B	C	D	E	G	H	I	J	K	L	M	N	O	P	Q	R	N Hg^a^
**Bardigiano**	27.1	8.3				18.8		12.5		4.2	16.7	2.1	4.2		2.1	4.2		**10**
**Monterufolino**				1.0							53.3		6.7	1.0		13.3	6.7	**6**
**Maremmano**	7.7	14.3	2.2	1.1	3.3	15.6	1.1	4.4	1.1		20.9	13.2	2.2	1.1	4.4	5.5	1.1	**16**
**Tolfetano**	11.5	11.5			7.7	15.4		7.7	7.7		30.8	3.8			3.8			**9**
**Lipizzan**		2.0	6.7			16.7					3.0	2.0				6.7		**6**
**Esperia**					17.9			21.4			32.1	10.7	10.7	7.1				**6**
**Murgese**	3.1		3.1	3.1	3.1	25.0			3.1		31.3	3.1			6.3	18.8		**10**
**Sardinian Anglo-Arab**	1.9	3.7			11.1	16.7		14.8			42.6	7.4	1.9					**8**
**Giara**						56.4		20.5			15.4	7.7						**4**
**Sarcidano**						26.7		40.0			10.0	23.3						**4**
**Italian Breeds**	6.1	6.9	1.2	1.2	4.2	19.4	0.2	11.3	1.0	0.5	27.2	9.3	2.5	1.5	2.0	4.7	0.7	**17**
**Arabian horse**		13.9	2.8			19.4		13.9	2.8			2.8	5.6	27.8	5.6	5.6		**10**
**Total**	5.6	7.4	1.4	1.1	3.8	19.4	0.2	11.5	1.1	0.5	25.0	8.8	2.7	3.6	2.3	4.7	0.7	**17**

^a^ Total number of haplogroups for each breed. Haplogroup affiliation is according to Achilli et al. [19].

The second most common haplogroup was G (19.4%) with the highest values in Giara (56.4%) and Sarcidano (26.7%), followed by I (11.3%), which peaks in Sarcidano (40.0%), followed by Giara (20.5%) and Esperia ponies (21.4%). According to the literature, haplogroups G and I should be more common in Asia and the Middle East, respectively [19]. The highest number of haplogroups was identified in the Maremmano breed (N = 16), followed by Bardigiano (N = 10) and Murgese (N = 10). As for the “insular” stocks, Giara and Sarcidano present only the major haplogroups (G, I, L, and M), while Sardinian Anglo-Arab displays a wider range of haplogroups, including A (1.9%), B (3.7%), E (11.1%) and N (1.9%). These data confirm the close genetic relationships among the Sardinian horse populations, especially between the Sarcidano and Giara breeds that share the same haplogroups and often the same haplotypes, as displayed in the presented network (Fig 2). Such a reconstructed network, based only on local Italian breeds and control-region data, allowed to date the mtDNA haplogroups to very ancient times (Table 5).

**Table 5:** MtDNA haplogroup ages based only on Italian control-region data.

Hg	N	N haplotypes	Rho estimate	Sigma	T (ka)	ΔT
**A**	25	6	1.84	1.02	10.2	5.6
**B**	33	10	2.96	1.35	16.4	7.5
**C**	6	5	1.40	0.72	7.8	4.0
**D**	5	3	0.80	0.40	4.4	2.2
**E**	17	4	0.29	0.16	1.6	0.9
**G**	86	21	2.51	0.22	13.9	1.2
**H**	1	1	n.a.	n.a.	n.a.	n.a.
**I**	51	10	3.00	1.38	16.6	7.6
**J**	5	4	1.25	0.75	6.9	4.2
**K**	2	1	n.a.	n.a.	n.a.	n.a.
**L**	111	25	3.49	0.18	19.3	1.0
**M**	39	11	3.29	1.48	18.2	8.2
**N**	12	3	0.40	0.24	2.2	1.4
**O**	16	4	1.00	0.62	5.5	3.5
**P**	10	6	0.75	0.53	4.2	2.9
**Q**^a^	21	13	2.95	0.52	16.3	2.9

^a^ Calculated by hand.

**Fig 3:**
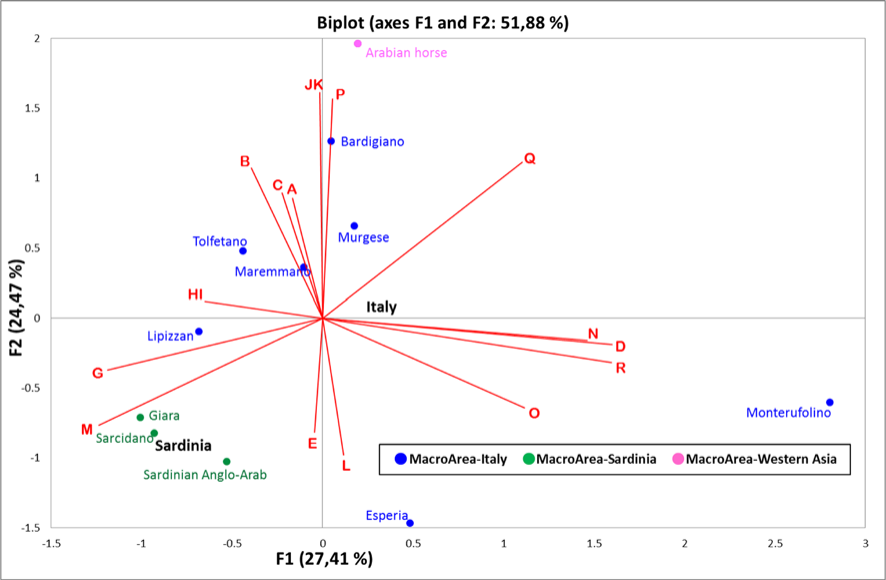
A two-dimensional breed-based bi-plot of mtDNA haplogroup profiles (S2 Table) from the eleven breeds analyzed in this study. The rarest haplogroups (with overall frequencies ≤ 0.5%) H and K were phylogenetically grouped with the corresponding sister clades I and J, respectively. The geographic labels, indicated in bold, represent the centroids of breeds typical of Italy (in blue) and Sardinia (in green).

In order to graphically display (and summarize) the mitochondrial relationships among the analyzed breeds, we performed a principal component analysis (PCA)–a method that considers each haplogroup as a discrete variable and allows a summary of the initial dataset into principal components (PCs). After variables reduction to PCs (haplogroup frequencies based on different haplotypes, S2 Table), the coordinates of the observations for the eleven populations were reported in a two-dimensional plot representing the horse genetic landscape of Italy (Fig 3).

The outlier position of Monterufolino is confirmed particularly along the first PC, while the second PC splits the Arabian horses from the other breeds. Moreover, Sardinian breeds clearly separate from Italian ones as also shown by the centroids (the centroid is the geometric center of a two-dimensional shape, as depicted here by breeds typical of a certain macro-geographic area, and it is calculated as the arithmetic average position of all points/breeds). It is well known that the mtDNA inheritance might be influenced by major stochastic processes, which in turn can be amplified by local bottlenecks and founder effects. Actually, the gene pools of geographically isolated populations are dramatically shaped by initial founding events (particularly in a uniparental system such as the mtDNA) that usually lead to low level of within-population genetic distances, as those reported for Giara and Sarcidano by both the PCA and the AMOVA (Table 3), in agreement with some previous studies [31]. The ostensible partial disagreement with the results reported by Morelli et al. [29], which considered Giara and Sarcidano as two distinct gene pools, could reside in the absence of two of the four haplogroups (I and M) shared by our Giara and Sarcidano samples. Moreover, we identified six different haplotypes shared by Giara and Sarcidano horses (one restricted only to these two breeds), which sum up to 84% of total samples (58 out of 69; S1 Table and Fig 2).

**Fig 4:**
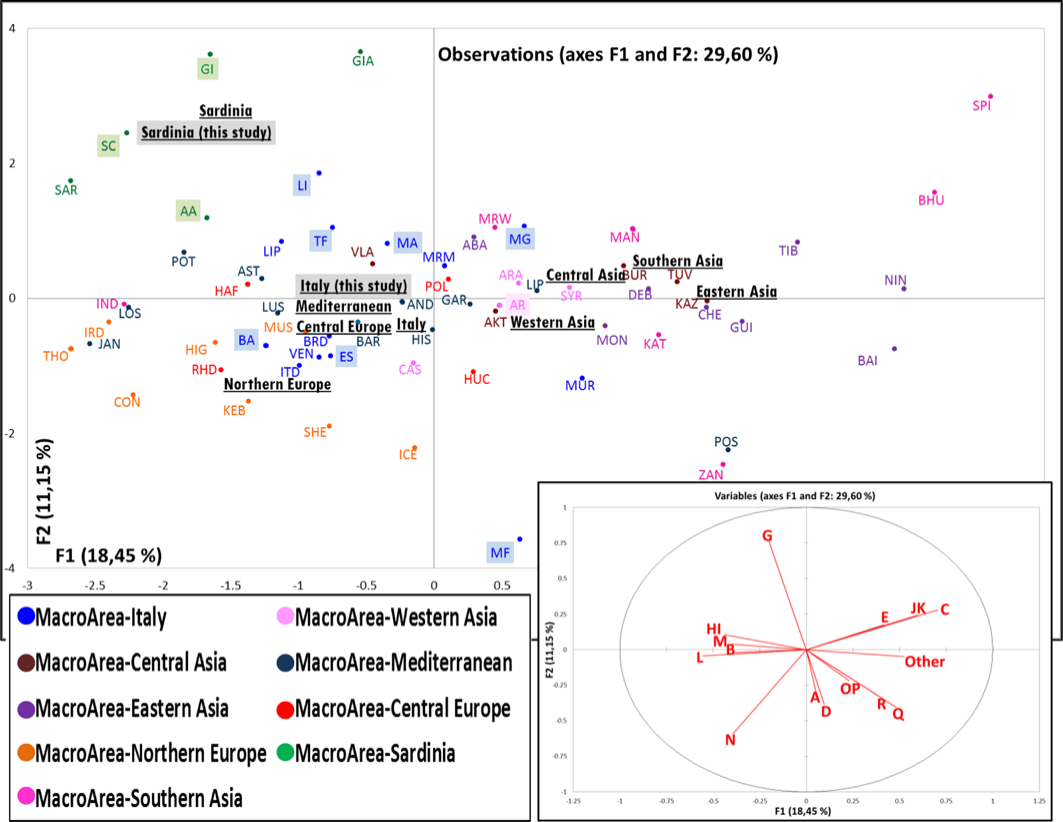
A two-dimensional region-based PCA plot obtained by including the available horse mtDNA data (S3 and S4 Tables). The eleven breeds analyzed in this study (and corresponding macroareas) are highlighted. The macrogeographic labels, indicated in bold and underlined, represent the centroids of breeds from the area. Only those breeds with at least 15 different haplotypes were considered statistically significant and included in the final PC analysis. Below is the plot of the contribution of each haplogroup to the first and second PC (projections of the axes of the original variables).

In order to determine whether the overall haplogroup frequencies in the Italian horse populations were indeed different from those of other populations worldwide, we repeated the PCA by including other GenBank data (S3 and S4 Tables). The overall plot, depicted by PCs 1 and 2 (Fig 4) confirms the outlier position of Monterufolino and the Sardinian horses, but at the same time highlights an overall geographic pattern from Northern Europe to Eastern Asia, as shown by the centroids position of each macrogeographic area.

The Italian breeds stand in an intermediate position together with most of the other Mediterranean stocks. The only notable exceptions are represented by the Bardigiano, which shows possible influences from Northern Europe, and particularly by the Murgese that seems to be closely related to the Asian breeds.

### The mtDNA peculiarities of some Italian breeds

A strong founder effect is evident in Monterufolino, the only Italian breed with a haplotype diversity lower than 0.8 and placed in an outlier position in both the Italian and the Eurasian population contexts (Figs 3 and 4). Such a peculiar gene pool could be easily connected to the breed’s history. In the nineties, its total population counted less than ten individuals [34] and we were able to identify the considerable number of seven distinctive founding mares.

The PCA analysis also revealed a peculiar localization of the Bardigiano pony within a Northern European genetic context, which was never reported in previous analyses (Sabbioni et al. 2005) [39]. This uniqueness among the Italian breeds could be explained by both its phenotype and its history. The Bardigiano is considered indigenous of Italy [34], but its origin could be traced back to the horses ridden by northern invaders during their incursions into the Italian Peninsula in the V century [40]. This original maternal legacy survived the recent dilution process due to the introduction of a diverse range of stallions from various breeds after World War II, especially *Franches Montagnes*.

Another peculiar position among Western Asian breeds is occupied by the Murgese horse, an ancient breed originated in Apulia during the Spanish domination (XVI-XVIII centuries). It is thought that the breed was developed by crossing a Spanish stock (partially Arab) with native horses, which share the same origin with the Neapolitan horse. Afterward a strict selection began in the early nineties and probably some matrilines from abroad were introduced. We identified 21 different haplotypes from the 46 presumed founding mares and based on our data they were mostly brought from Asia.

A further interesting finding is the clear separation between the Lipizzan horses from Italy and those from abroad (Fig 4). The Lipizzan breed dates back to the XVI century, when it was bred at Lipica (now in Slovenia). In the following centuries several maternal lines have been developed from eight traditional Lipizzan studs [4, 41]. Strict breeding rules were followed to keep separate different genetic reserves as demonstrated from the above mentioned peculiar PCA position of the Lipizzan horses from the Italian breeding farm of Monterotondo, whose eleven founding maternal lines are completely represented by the eleven different haplotypes reported in S1 Table.

## Conclusion

Besides confirming a widespread mitochondrial variability in Italy, as already reported [29, 31, 32], this study provides a more comprehensive reassessment of the mitochondrial genetic relationships among ten typical Italian hotblood/warmblood horse and pony breeds. The different mtDNA haplotypes are not preferentially distributed among breeds. The only significant haplotype-based population structure was recognized when considering as a possible differentiation factor the (geographic) isolation of the Monterufolino and Sardinian breeds. The same four haplogroups were identified in the Giara and Sarcidano breeds (often along with the same haplotypes), whose mitochondrial similarities were confirmed in a wider Eurasian context through the PC analysis. The outcoming mtDNA genetic landscape of Eurasia shows a clear geographic pattern and highlights a group of closely related intermediate breeds mostly from the Italian Peninsula. This genetic feature likely reflects the geographic position of Italy, in the center of the Mediterranean Sea, and its cultural/economic past as a crossroad of migratory waves from the Western Asian coasts to Continental Europe. It is worth nothing that Italian breeds show a frequency of haplogroup L (23.9%) which is intermediate between those recorded in Western Asia (18.1%) and in Continental Europe (31.1%) (S5 Table). Moreover, an additional clue of a putative east-west direction of the gene flow is given by the overall haplogroup frequencies of Italian horses, which are somehow more similar to the breeds from South-West Asia (χ^2^: 27.5; *p-value*: 0.006) than to those from Continental Europe (χ^2^: 74.8; *p-value*: <0.001), as already indicated [32]. These findings probably reflect the overall mtDNA legacy of the ancestral mares (of eastern origins) that long time ago (see age estimates in Table 5) were probably used at the initial stages of breeding selections. Those mitochondrial lineages were also preserved during the final establishment of pure breeds that was mainly reached through sex-biased breeding practices [42], which often involved the intensive use of few selected external stallions [43, 44]. Thus, the impact on the original mtDNA gene pool could have been marginal, as also testified by the only four haplotypes shared between the Arabian horses and the ten Italian breeds here analyzed in spite of the well-recognized use of the Arabian stallions to revitalize some Italian breeds. As for the recent times, our mtDNA data lend also genetic support to some historical theories about the origin of some Italian breeds.

In conclusion, we confirm that the mitogenome is an appropriate resource in studies aiming to reconstruct the maternal ancestral origins of local breeds and to evaluate genetic continuity with the original stocks.

## Materials and Methods

### Ethics statement

All experimental procedures were reviewed and approved by the Animal Research Ethics Committee of the Universities of Perugia and Pavia in accordance with the European Union Directive 86/609.

### Sample collection

DNA was extracted from 1,2 ml of peripheral blood samples of 407 specimens belonging to ten Italian native breeds: Sardinian Anglo-Arab (Anglo-Arabo Sardo, AA; n = 54), Bardigiano (BA; n = 48), Esperia (ES; n = 28), Giara (GI; n = 39), Lipizzan (Lipizzano, LI; n = 30), Maremmano (MA; n = 90), Monterufolino (MF; n = 30), Murgese (MG; n = 32), Sarcidano (SC; n = 30), Tolfetano (TF; n = 26). Also 36 samples of Arabian horses were included (Arabo, AR; n = 36). Horses were sampled from different Italian regions: Emilian Appennines, Latium, Apulia, Tuscany, Sardinia (Fig 1). Overall, 266 were females, 112 were males and six were geldings; no gender information was available for 59 specimens.

For the ten Italian breeds analyzed in this study, genealogical data are recorded in Studbooks (Bardigiano, Lipizzan, Maremmano, Murgese and Sardinian Anglo-Arab) or Anagraphic Registers (Esperia, Giara, Monterufolino, Sarcidano and Tolfetano). Genealogical information was considered, when available (i.e. for Lipizzan, Sardinian Anglo-Arab, Maremmano and Murgese), in order to select unrelated animals, while all other breeds (Bardigiano, Esperia, Giara, Monterufolino, Sarcidano and Tolfetano) were randomly sampled.

Total DNA was extracted from blood samples by automated extraction using the MagCore® Automated Nucleic Acid Extractor, following the provided protocol.

### PCR amplification and sequencing of the mtDNA control region

For all animals, the mtDNA region comprised between nps 15364 and 563 was amplified by using the following oligonuclotides: forward 5’-AAACCAGAAAAGGGGGAAAA-3’; reverse 5’-TGGCGAATAGCTTTGTTGTG-3’. Oligonucleotides were designed employing the GenBank published Equine Reference Sequence (ERS) NC001640 (derived from X79547) [45]. The PCR fragment of 1192 bp encompassing the entire mtDNA control region (15469–16660) was purified using exonuclease I and alkaline phosphatase (ExoSAP-IT® enzymatic system-USB Corporation, Cleveland, OH, USA) and then sent to BMR-Genomics srl (www.bmrgenomics.com) for Sanger sequencing with the primer forward 5’-CACCCAAAGCTGAAATTCTA-3’.

### Mitochondrial DNA sequence analyses

Sequences (610 bps from np 15491 to np 16100) were assembled and aligned to ERS using Sequencher™ 5.10 (Gene Codes Corporation). Whenever electropherograms showed ambiguities, new PCR amplifications and sequencing reactions were performed. All mtDNA D-loop sequences determined in this study were deposited in GenBank with accession numbers KU711082-KU711507.

Several mtDNA sequence variation parameters were estimated by using DnaSP 5.1 software [46]. Analysis of MOlecular VAriance (AMOVA) and pairwise Fst calculations were performed using the Arlequin v. 3.5 software package [47]. The statistical significance of the values was estimated by permutation analysis using 100 replications. Intra- as well as inter-population comparisons were performed based on the number of pairwise differences between sequences and figured using an Arlequin integrated R script (http://www.rproject.org/).

The evolutionary relationships among haplotypes were visualized through the construction of different median-joining networks using Network 4.6 (www.fluxusengineering.com), one for each haplogroup (C, D, E, G, L, Q, and R) and macro-haplogroup (A’B, H’I, J’K, M’N, and O’P), then parsimoniously connected by hand according to mutational diagnostic motifs identified by Achilli et al. [19]. The evolutionary distances were computed as averaged distance (ρ) of the haplotypes within a clade from the respective root haplotype, accompanied by a heuristic estimate of SE (σ). All positions containing gaps and ambiguous data were eliminated from the dataset. Estimate of the time to the most recent common ancestor for each cluster was calculated using a corrected age estimate of about 2.96 x 10^−7^ per nucleotide per year in the whole control region [19], which corresponds to 5,540 years per substitution over the sequenced region of 610 bps.

Principal component analyses (PCA) were performed using Excel software implemented by XLSTAT, as described elsewhere [48]. Two PCA were carried out one by considering only our sample; the other by including the available horse mtDNA records obtained from GenBank. The PCA is a widely used dimension-reduction method which seeks to explain the variance of multivariate data by a smaller number of variables (the principal components, PCs), which are linear functions of the original variables, which in this case are the haplogroup frequencies. Considering the high degree inbreeding, which mostly characterizes common selection strategies, the haplogroup frequencies used as source data for the PCA were calculated by considering only different haplotypes within the same breed. The rarest haplogroups were phylogenetically grouped and among the large plethora of available data, only those represented by at least 15 different haplotypes were included in the analysis in order to increase the statistical significance. After having reduced the variables (haplogroups) to PCs, we reported the coordinates of the observations (breeds here and elsewhere analyzed) in two-dimensional graphics representing the genetic landscape of Italy and West Eurasia.

## Supporting Information

S1 Fig Plot of pairwise population genetic distances obtained by the concomitant analysis of all Italian breeds. Breed code as in Table 2.
(TIF)

S1 Table Control-region haplotypes and haplogroup classification of the 443 horse mtDNAs from Italian breeds (n = 407) and Arabian horses (n = 36). (XLSX)

S2 Table Source data for the PCA of ten Italian breeds and Arabian horses here analyzed. Haplogroup frequencies are calculated on different haplotypes.
(XLSX)

S3 Table Source data for the PCA of Eurasian breeds. Haplogroup frequencies are calculated on different haplotypes.
(XLSX)

S4 Table A summary of the available horse mtDNA data. (DOCX)

S5 Table A geographic comparison of haplogroup frequencies (%). (DOCX)

## References

[1] Dovc P, Kavar T, Solkner H, Achmann R. Development of the Lipizzan horse breed. Reprod Domest Anim. 2006;41(4):280–5. 10.1111/j.1439-0531.2006.00726.x . 16869882

[2] Hill EW, Bradley DG, Al-Barody M, Ertugrul O, Splan RK, Zakharov I, et al History and integrity of thoroughbred dam lines revealed in equine mtDNA variation. Anim Genet. 2002;33(4):287–94. 12139508 10.1046/j.1365-2052.2002.00870.x

[3] Jansen T, Forster P, Levine MA, Oelke H, Hurles M, Renfrew C, et al Mitochondrial DNA and the origins of the domestic horse. Proc Natl Acad Sci U S A. 2002;99(16):10905–10. 10.1073/pnas.152330099 12130666 PMC125071

[4] Kavar T, Brem G, Habe F, Solkner J, Dovc P. History of Lipizzan horse maternal lines as revealed by mtDNA analysis. Genet Sel Evol. 2002;34(5):635–48. 10.1051/gse:2002028 12427390 PMC2705438

[5] Lopes MS, Mendonca D, Cymbron T, Valera M, da Costa-Ferreira J, Machado Ada C. The Lusitano horse maternal lineage based on mitochondrial D-loop sequence variation. Anim Genet. 2005;36(3):196–202. 10.1111/j.1365-2052.2005.01279.x . 15932397

[6] Royo LJ, Alvarez I, Beja-Pereira A, Molina A, Fernandez I, Jordana J, et al The origins of Iberian horses assessed via mitochondrial DNA. J Hered. 2005;96(6):663–9. 10.1093/jhered/esi116 . 16251517

[7] McGahern A, Bower MA, Edwards CJ, Brophy PO, Sulimova G, Zakharov I, et al Evidence for biogeographic patterning of mitochondrial DNA sequences in Eastern horse populations. Anim Genet. 2006;37(5):494–7. 10.1111/j.1365-2052.2006.01495.x . 16978180

[8] Moridi M, Masoudi AA, Vaez Torshizi R, Hill EW. Mitochondrial DNA D-loop sequence variation in maternal lineages of Iranian native horses. Anim Genet. 2013;44(2):209–13. 10.1111/j.1365-2052.2012.02389.x . 22732008

[9] Khanshour AM, Cothran EG. Maternal phylogenetic relationships and genetic variation among Arabian horse populations using whole mitochondrial DNA D-loop sequencing. BMC Genet. 2013;14:83 10.1186/1471-2156-14-83 24034565 PMC3847362

[10] Bowling AT, Del Valle A, Bowling M. A pedigree-based study of mitochondrial D-loop DNA sequence variation among Arabian horses. Anim Genet. 2000;31(1):1–7. 10690354 10.1046/j.1365-2052.2000.00558.x

[11] Głażewska I. Speculations on the origin of the Arabian horse breed. Livest Sci. 2010;129:49–55.

[12] Cothran EG, Juras R, Macijauskiene V. Mitochondrial DNA D-loop sequence variation among 5 maternal lines of the Zemaitukai horse breed. Genet Mol Biol. 2005; 28(4):677–81.

[13] Glazewska I, Wysocka A, Gralak B, Sell J. A new view on dam lines in Polish Arabian horses based on mtDNA analysis. Genet Sel Evol. 2007;39(5):609–19. 10.1051/gse:2007025 . 17897600 PMC2682809

[14] Guastella AM, Zuccaro A, Criscione A, Marletta D, Bordonaro S. Genetic analysis of Sicilian autochthonous horse breeds using nuclear and mitochondrial DNA markers. J Hered. 2011;102(6):753–8. 10.1093/jhered/esr091 . 21914666

[15] Ivanković A, Ramljak J, Konjačić M, Kelava N, Dovč P, Mijić P. Mitochondrial D-loop sequence variation among autochthonous horse breeds in Croatia. Czech J Anim Sci. 2009;54(3):101–11.

[16] Achilli A, Olivieri A, Pellecchia M, Uboldi C, Colli L, Al-Zahery N, et al Mitochondrial genomes of extinct aurochs survive in domestic cattle. Curr Biol. 2008;18(4):R157–8. 10.1016/j.cub.2008.01.019 . 18302915

[17] Colli L, Lancioni H, Cardinali I, Olivieri A, Capodiferro MR, Pellecchia M, et al Whole mitochondrial genomes unveil the impact of domestication on goat matrilineal variability. BMC genomics. 2015;16(1):1115 10.1186/s12864-015-2342-2 . 26714643 PMC4696231

[18] Lancioni H, Di Lorenzo P, Ceccobelli S, Perego UA, Miglio A, Landi V, et al Phylogenetic relationships of three Italian merino-derived sheep breeds evaluated through a complete mitogenome analysis. PLoS One. 2013;8(9):e73712 10.1371/journal.pone.0073712 24040036 PMC3767607

[19] Achilli A, Olivieri A, Soares P, Lancioni H, Hooshiar Kashani B, Perego UA, et al Mitochondrial genomes from modern horses reveal the major haplogroups that underwent domestication. Proc Natl Acad Sci U S A. 2012;109(7):2449–54. 10.1073/pnas.1111637109 22308342 PMC3289334

[20] Vilstrup JT, Seguin-Orlando A, Stiller M, Ginolhac A, Raghavan M, Nielsen SC, et al Mitochondrial phylogenomics of modern and ancient equids. PLoS One. 2013;8(2):e55950 10.1371/journal.pone.0055950 23437078 PMC3577844

[21] Lippold S, Knapp M, Kuznetsova T, Leonard JA, Benecke N, Ludwig A, et al Discovery of lost diversity of paternal horse lineages using ancient DNA. Nat Commun. 2011;2:450 10.1038/ncomms1447 . 21863017

[22] Warmuth V, Eriksson A, Bower MA, Barker G, Barrett E, Hanks BK, et al Reconstructing the origin and spread of horse domestication in the Eurasian steppe. Proc Natl Acad Sci U S A. 2012;109(21):8202–6. 10.1073/pnas.1111122109 22566639 PMC3361400

[23] Outram AK, Stear NA, Bendrey R, Olsen S, Kasparov A, Zaibert V, et al The earliest horse harnessing and milking. Science. 2009;323(5919):1332–5. 10.1126/science.1168594 . 19265018

[24] Bigi D, Zanon A. Atlante delle razze autoctone italiane: Bovini, Equini, Ovicaprini, Suini allevati in Italia. Milano2008.

[25] Pieragostini E, Rizzi R, Bramante G, Perrotta G, Caroli A. Genetic study of Murgese horse from genealogical data and microsatellites. Ital J Anim Sci. 2005;4:197–202.

[26] Felicetti M, Lopes MS, Verini-Supplizi A, Machado Ada C, Silvestrelli M, Mendonca D, et al Genetic diversity in the Maremmano horse and its relationship with other European horse breeds. Anim Genet. 2010;41 Suppl 2:53–5. 10.1111/j.1365-2052.2010.02102.x . 21070276

[27] Maretto F, Mantovani R. Genetic variability of Italian Heavy Draught Horse. Ital J Anim Sci. 2009;8(3):95–7

[28] Bigi D, Perrotta G. Genetic structure and differentiation of the Italian catria horse. J Hered. 2012;103(1):134–9. 10.1093/jhered/esr121 . 22156056

[29] Morelli L, Useli A, Sanna D, Barbato M, Contu D, Pala M, et al Mitochondrial DNA lineages of Italian Giara and Sarcidano horses. Genet Mol Res. 2014;13(4):8241–57. 10.4238/2014.October.20.1 . 25366719

[30] Zuccaro A, Bordonaro S, Guastella AM, Longeri M, Cozzi MC, Guastella AM, et al Mitochondrial DNA control region variation in Sanfratellano horse and two other Sicilian autochthonous breeds. Ital J Anim Sci. 2009;8(2):180–2

[31] Cozzi MC, Strillacci MG, Valiati P, Bighignoli B, Cancedda M, Zanotti M. Mitochondrial D-loop sequence variation among Italian horse breeds. Genet Sel Evol. 2004;36(6):663–72. 10.1051/gse:2004023 15496286 PMC2697199

[32] Bigi D, Perrotta G, Zambonelli P. Genetic analysis of seven Italian horse breeds based on mitochondrial DNA D-loop variation. Anim Genet. 2014;45(4):593–5. 10.1111/age.12156 . 24702170

[33] http://www.aia.it/ [Web Site]. 2015.

[34] http://dad.fao.org/ [Web Site]. 2014.

[35] Yue XP, Qin F, Campana MG, Liu DH, Mao CC, Wang XB, et al Characterization of cytochrome b diversity in Chinese domestic horses. Anim Genet. 2012;43(5):624–6. 10.1111/j.1365-2052.2011.02298.x 22497593

[36] Kakoi H, Tozaki T, Gawahara H. Molecular analysis using mitochondrial DNA and microsatellites to infer the formation process of Japanese native horse populations. Biochem Genet. 2007;45(3–4):375–95. 10.1007/s10528-007-9083-0 . 17265183

[37] Cieslak M, Pruvost M, Benecke N, Hofreiter M, Morales A, Reissmann M, et al Origin and history of mitochondrial DNA lineages in domestic horses. PLoS One. 2010;5(12):e15311 10.1371/journal.pone.0015311 21187961 PMC3004868

[38] Alvarez I, Fernandez I, Cuervo M, Martin D, Lorenzo L, Goyache F. Short communication. Mitochondrial DNA diversity of the founder populations of the Asturcón pony. Spanish Journal of Agricultural Research. 2013;11(3):702 10.5424/sjar/2013113-4127

[39] Di Stasio L, Perrotta G, Blasi M, Lisa C. Genetic characterization of the Bardigiano horse using microsatellite markers. Ital J Anim Sci. 2008;7:243–50.

[40] Bongianni M. Simon & Schuster's Guide to Horses & Ponies of the World Simon & Schuster Building, New York: Simon & Schuster Inc., New York; 1988.

[41] Zechner P, Sölkner J, Bodo I, Druml T, Baumung R, Achmann R, et al Analysis of diversity and population structure in the Lipizzan horse breed based on pedigree information. Livest Prod Sci. 2002;77(2–3):137–46. 10.1016/S0301-6226(02)00079-9.

[42] Vila C, Leonard JA, Gotherstrom A, Marklund S, Sandberg K, Liden K, et al Widespread origins of domestic horse lineages. Science. 2001;291(5503):474–7. 10.1126/science.291.5503.474 . 11161199

[43] Lindgren G, Backstrom N, Swinburne J, Hellborg L, Einarsson A, Sandberg K, et al Limited number of patrilines in horse domestication. Nat Genet. 2004;36(4):335–6. 10.1038/ng1326 . 15034578

[44] Wallner B, Vogl C, Shukla P, Burgstaller JP, Druml T, Brem G. Identification of genetic variation on the horse y chromosome and the tracing of male founder lineages in modern breeds. PLoS One. 2013;8(4):e60015 10.1371/journal.pone.0060015 23573227 PMC3616054

[45] Xu X, Arnason U. The complete mitochondrial DNA sequence of the horse, *Equus caballus*: extensive heteroplasmy of the control region. Gene. 1994;148(2):357–62. 7958969 10.1016/0378-1119(94)90713-7

[46] Librado P, Rozas J. DnaSP v5: a software for comprehensive analysis of DNA polymorphism data. Bioinformatics. 2009;25(11):1451–2. 10.1093/bioinformatics/btp187 19346325

[47] Excoffier L, Laval G, Schneider S. Arlequin (version 3.0): an integrated software package for population genetics data analysis. Evol Bioinform Online. 2005;1:47–50 PMC2658868 19325852

[48] Achilli A, Olivieri A, Pala M, Metspalu E, Fornarino S, Battaglia V, et al Mitochondrial DNA variation of modern Tuscans supports the near eastern origin of Etruscans. Am J Hum Genet. 2007;80(4):759–68. 10.1086/512822 17357081 PMC1852723

