# Supplementary figures and images for: An Overview of Ten Italian Horse Breeds through Mitochondrial DNA
Source: PLoS One. 2016 Apr 7;11(4):e0153004. doi: 10.1371/journal.pone.0153004 (PMC4824442; doi:10.1371/journal.pone.0153004)

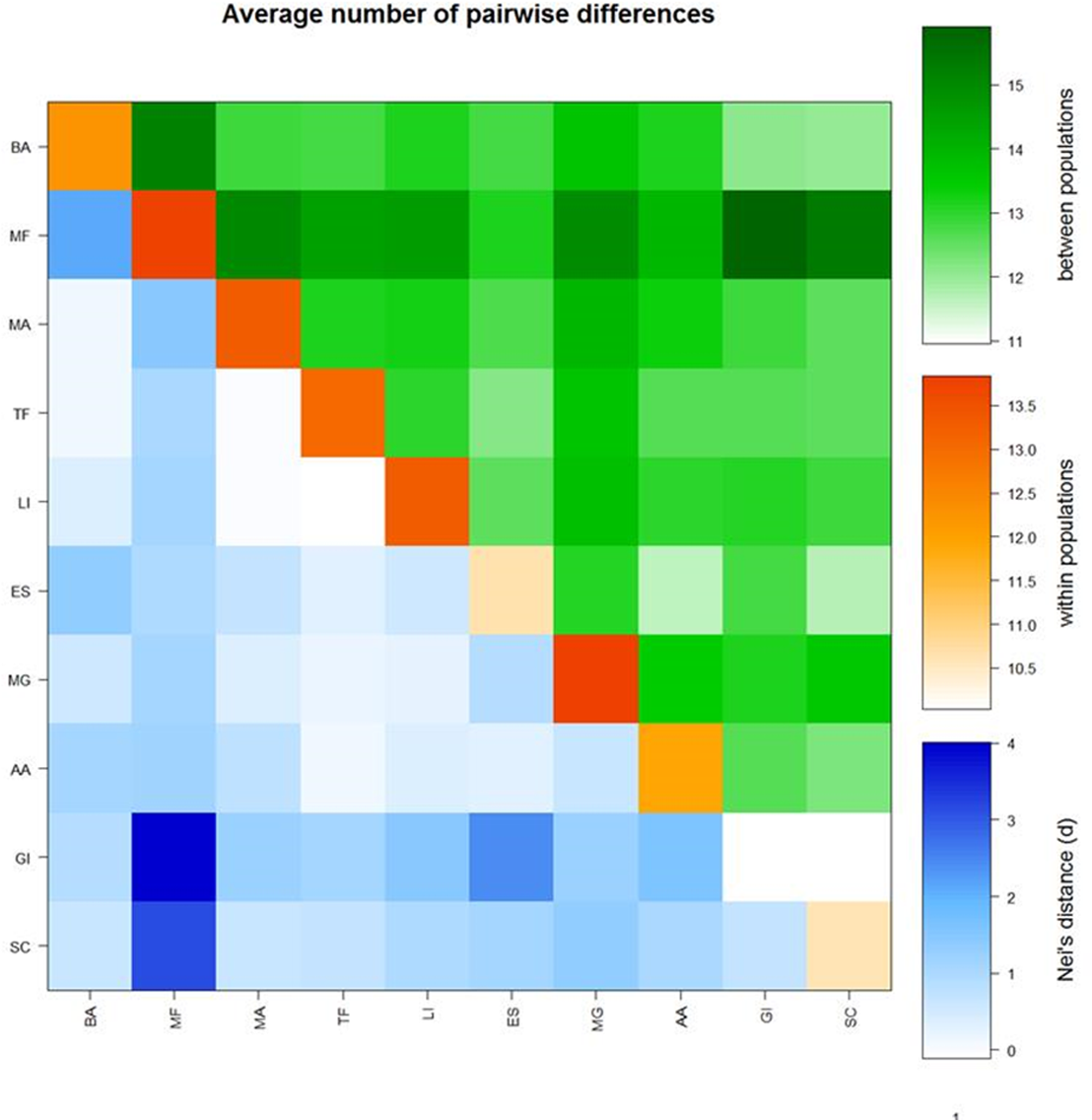

Supplement: S1 Fig — Breed code as in Table 2. (TIF) [file pone.0153004.s001.tif]
