# Supplementary material for: An Overview of Ten Italian Horse Breeds through Mitochondrial DNA
Source: PLoS One. 2016 Apr 7;11(4):e0153004. doi: 10.1371/journal.pone.0153004 (PMC4824442; doi:10.1371/journal.pone.0153004)
Supplement: S4 Table — (DOCX) [file pone.0153004.s005.docx]

**S4 Table.** **A summary of the available horse mtDNA data.**

| Macroarea^a^ | Breed^b^ | Code | Total | References and Accession numbers |
| --- | --- | --- | --- | --- |
| East Asia | Abaga | ABA | 19 | GU561998-GU561999; JN224838-JN224854 |
|  | Baise Pony (Guangxi) | BAI | 20 | DQ297634; GQ203128-GQ203144; GQ222059-GQ222060 |
|  | Cheju | CHE | 80 | [1, 2]; AY246201-AY246208 |
|  | Debao Pony | DEB | 25 | [3]; EU826536; FJ392562-FJ392580; FJ502833; GQ203125-GQ203127 |
|  | Guan Mountain | GUM | 10 | [4] |
|  | Guanzhong | GUA | 27 | [5]; AY136785-AY136786 |
|  | Guizhou | GUI | 95 | [5]; JQ710902-JQ710934 |
|  | Mongolian | MON | 146 | [1, 4, 6-9]; AF072996; AP013087-AP013093; DQ297622-DQ297633; JQ936335-JQ936405; KF038165-KF038166 |
|  | Ningqiang | NIN | 30 | [5]; FJ502832; FJ502839; HQ845202 |
|  | Przewalskii | PRZ | 16 | [1, 10-14]; AF072994-AF072995; HQ439484; KC143355 |
|  | Tibetan Pony | TIB | 16 | DQ986464-DQ986479 |
|  | Wuzhumuqin (Ujumqin) | WUZ | 21 | GU561995-GU561997; JN224855-JN224866; JN790897-JN790902 |
|  | Noma | NOM | 14 | [7] |
|  | Misaki | MIS | 22 | [7] |
|  | Yonaguni | YON | 29 | [7] |
|  | Taishu Pony (Tsushima) | TAI | 16 | [7] |
|  | Hokkaido Pony | HOK | 34 | [7]; AP013081-AP013086 |
|  | Tokara Pony | TOK | 19 | [7] |
|  | Kiso | KIS | 12 | [7] |
|  | Misaki | MIS | 4 | [7] |
| Central Asia | Akhal-Teke | AKT | 57 | [4, 10, 15]; AY246174-AY246179; HQ439441-HQ439442 |
|  | Kazakh | KAZ | 19 | [5]; AF072985 |
| Northern Asia | Akhal-Teke | AKT | 4 | [15] |
|  | Buryat (Buryatskaya) | BUR | 22 | JQ936455-JQ936476 |
|  | Mesenskay | MES | 18 | [4] |
|  | Orlov | ORL | 19 | [4]; HQ439481 |
|  | Transbaikalian | TRB | 24 | JQ936431-JQ936454 |
|  | Tuva (Tuvinskaya) | TUV | 36 | [16]; JQ936406-JQ936430 |
|  | Vladimir Heavy Draught | VLA | 21 | HQ439500; KC847166; KF849272-KF849290 |
|  | Vyatskaya | VYA | 19 | [4]; HQ439495 |
|  | Yakut (Yakutskaya) | YAK | 21 | [4]; HQ439467 |
| Southern Asia | Bhutia Pony | BHU | 24 | HE565672-HE565695 |
|  | Indian Thoroughbred | IND | 23 | HE575410-HE575432 |
|  | Kathiawari | KAT | 23 | HE580440-HE580462 |
|  | Manipuri Pony | MAN | 69 | [17, 18]; HE565867-HE565889 |
|  | Marwari | MRW | 24 | HE572595-HE572619 |
|  | Spiti Pony | SPI | 16 | HE565598-HE565610; HE572592-HE572594 |
|  | Zaniskari Pony | ZAN | 24 | HE565647-HE565670 |
| Middle East | Anatolian | ANA | 15 | [16] |
|  | Arabian horse | ARA | 115 | [6, 10, 19, 20]; This study; AF064627-AF064629; AF072989; AP013078; AY246180-AY246185; HQ439447-HQ439449; HQ439488 |
|  | Caspian | CAS | 31 | [6, 8-10]; AY246195-AY246200 |
|  | Cukorova | CUK | 12 | [16] |
|  | Davenport | DAV | 18 | [8] |
|  | Saudi | SAU | 72 | [8] |
|  | Syrian | SYR | 120 | [8] |
|  | Unspecified Iranian Breed | UIB | 14 | [10] |
|  | USA Saudi | USU | 27 | [8] |
| Balkans | Croatian Coldbloods | CRC | 20 | EF494073-EF494083; EF495139; EF495144; EF495146 |
|  | Lipizzan | LIP | 37 | [21, 22] |
|  | Murinsulaner (Medjimurski konj) | MUR | 15 | EF494079-EF494081; EF495145-EF495151 |
|  | Posavina | POS | 27 | AF431965-AF431969; AY575127-AY575129; EF494073; EF495133-EF495143 |
| Northern Europe | Clydesdale | CLY | 17 | [9, 10]; AY246214-AY246218; HQ439455 |
|  | Connemara | CON | 24 | [9, 16] |
|  | Dales Pony | DAL | 12 | [9] |
|  | Dartmoor Pony | DAR | 12 | [9] |
|  | Eriskay | ERI | 12 | [9] |
|  | Exmoor Pony | EXM | 32 | [6, 9, 10]; AF072992-AF072993; AY246219-AY246224 |
|  | Fell Pony | FEL | 28 | [9, 23]; AF072981-AF072982 |
|  | Fjord | FJR | 11 | [9] |
|  | Highland Pony | HIG | 11 | [9] |
|  | Icelandic | ICE | 489 | [6, 9, 10, 24]; AF072988; HQ439465-HQ439466; KJ741404-KJ741846 |
|  | Irish Draught | IRD | 59 | [4] |
|  | Kerry Bog Pony | KEB | 49 | [4, 9] |
|  | Norwegian Fjord | NRW | 13 | [6, 10]; HQ439463 |
|  | Scottish Highland | SCH | 31 | [6, 23] |
|  | Shetland Pony | SHE | 87 | [6, 9, 16, 23]; AF072977-AF072979; AY246253-AY246258; HQ439489 |
|  | Shire | SHI | 12 | [6, 10]; AF072975-AF072976; HQ439490 |
|  | Thoroughbred | THO | 222 | [7, 16, 23, 25, 26]; AF072990-AF072991; AP013100-AP013101; AY246266-AY246271; DQ297636; DQ324048; JN790873-JN790878; KJ917283-KJ917308 |
|  | Turks and Caicos | TUC | 11 | [9] |
|  | Welsh Pony | WEL | 16 | [6, 9]; AF072983; HQ439496-HQ439499 |
| Middle Europe | Belgian Draft | BEL | 13 | [10]; AF064630-AF064632; AY246186-AY246194 |
|  | Black Forest (Schwarzwalder Kaltblut) | BLA | 55 | HQ439487; KC147073-KC147126 |
|  | Camargue | CAM | 53 | HQ439454; KC147127-KC147178 |
|  | Comtois | COM | 25 | KC147179-KC147203 |
|  | Duelmener Pony | DUE | 10 | [6] |
|  | Einsiedler | EIN | 28 | KC147014-KC147041 |
|  | Franches-Montagnes (Freiberger) | FRA | 31 | KC147042-KC147072 |
|  | Haflinger | HAF | 41 | [9, 25, 27]; AP013080; AY246236-AY246241; HQ439464 |
|  | Hispano-Breton | HIB | 20 | [28] |
|  | Holstein | HOL | 13 | [6]; AY575111-AY575112; HQ439458 |
|  | Hucul | HUC | 29 | [15, 29]; AY575113-AY575114; KC143336-KC143352 |
|  | Lipizzan | LIP | 1 | [29] |
|  | Noriker (Noric) | NOR | 10 | [6]; AY246248-AY246252; HQ439480 |
|  | Rhineland Heavy Draft | RHD | 26 | [6]; HQ439485; HQ439491 |
|  | Senner | SEN | 19 | [6] |
|  | Trakehner | TRK | 10 | [6, 10]; AY575130-AY575132; HQ439493 |
|  | Percheron | PER | 12 | [7] |
|  | Polish Arabian | POL | 56 | [8, 30] |
| Mediterranean Area | Andalusian | AND | 30 | [6, 10, 20, 31, 32] |
|  | Asturcón Pony | AST | 148 | [17, 20, 32]; HQ827083-HQ827090; KC527020 |
|  | Bardigiano | BRD | 66 | [27]; This study |
|  | Caballo de Corro | CCO | 19 | [32]; HQ827099-HQ827103 |
|  | Carthusian | CAR | 10 | [32] |
|  | Esperia | ESP | 28 | This study |
|  | Garrano | GAR | 18 | [31, 32]; AY246232; AY246234 |
|  | Giara | GIA | 69 | [10, 25, 33]; This study |
|  | Hispano-Breton | HIS | 44 | EF014970-EF014989; [28] |
|  | Italian Heavy Draught | ITD | 27 | [27] |
|  | Italian Trotter | ITR | 5 | [25] |
|  | Jaca Navarra | JAN | 15 | HQ827104-HQ827118 |
|  | Lipizzan | LIP | 52 | [25, 27]; This study |
|  | Losina | LOS | 23 | HQ827119-HQ827129; [20]; [32] |
|  | Lusitano | LUS | 49 | [6, 31, 34]; AY246242-AY246247 |
|  | Mallorquina | MAL | 10 | [20]; HQ827130-HQ827135 |
|  | Maremmano | MRM | 120 | [10, 25, 27]; This study |
|  | Marismeno | MAR | 22 | [32]; HQ827136-HQ827145 |
|  | Menorquina | MEN | 10 | HQ827146-HQ827155 |
|  | Mérens Pony | MER | 12 | [32] |
|  | Monterufolino | MRF | 30 | This study |
|  | Murgese | MUR | 55 | [27]; This study |
|  | Pottok | POT | 21 | [20, 32]; HQ827156-HQ827161 |
|  | Pura Raza Espanola | PRE | 40 | [28, 31, 34] |
|  | Salernitano | SAL | 24 | [35] |
|  | Sanfratellano | SAN | 20 | EU569297; EU750717; EU750720; EU750722-EU750726; EU750729-EU750730; EU831234; EU831238 |
|  | Sarcidano | SAR | 53 | [25, 33]; This study |
|  | Sardinian Anglo-Arab | SAA | 54 | This study |
|  | Sicilian Indigenous | SII | 20 | EU604815-EU604817; EU750716; EU750718-EU750719; EU750721; EU750727-EU750728; EU750731; EU831235; EU831237; EU831239 |
|  | Sicilian Oriental Purebred | SOP | 20 | EU750729 |
|  | Sorraia | SOR | 30 | [6, 31, 36]; AY246259-AY246265; HQ827162-HQ827163 |
|  | Tolfetano | TLF | 26 | This study |
|  | Unspecified Italian Breed | ITA | 3 | [10] |
|  | Ventasso | VEN | 36 | [27] |
| Africa | Barb | BAR | 49 | [6]; EF686021-EF686045; HQ439453 |
|  | Fulani | FUL | 12 | [16] |
|  | USA Egyptian | UEG | 26 | [8] |
| Northern America | Canadian | CAN | 11 | [9] |
|  | Kiger Mustang | KIG | 27 | [6, 31] |
|  | Lac La Croix Indian Pony | LAC | 10 | [9] |
|  | Mustang | MUS | 36 | [6] |
|  | Newfoundland Pony | NFL | 17 | [9] |
|  | Sable Island Pony | SAB | 21 | [9] |
|  | Saint Pierre et Miquelon | SPM | 11 | [9] |
|  | Standardbred | STA | 13 | [9]; AF072984 |
|  | Sulphur Mustang | SUL | 27 | [6, 31]; AY997188-AY997189; AY997203-AY997204 |
| Southern America | Brazilian Criollo | BRA | 10 | [31] |
| Total |  |  | **4720** |  |

Sequences were inferred from both D-loop and complete mitogenomes recorded in GenBank.

^a^ Breeds were grouped in macroareas considering their geographic origin.

^b^ We considered only breeds with at least ten available samples; while Italian breeds were all included.

**Supporting References**

1. Kim KI, Yang YH, Lee SS, Park C, Ma R, Bouzat JL, et al. Phylogenetic relationships of Cheju horses to other horse breeds as determined by mtDNA D-loop sequence polymorphism. Anim Genet. 1999;30(2):102-8. PubMed PMID: 10376300.

2. Yang Z. PAML: a program package for phylogenetic analysis by maximum likelihood. Comput Appl Biosci. 1997;13(5):555-6. Epub 1997/11/21. PubMed PMID: 9367129.

3. Jiang Q, Wei Y, Huang Y, Jiang H, Guo Y, Lan G, et al. The complete mitochondrial genome and phylogenetic analysis of the Debao pony (Equus caballus). Mol Biol Rep. 2011;38(1):593-9. doi: 10.1007/s11033-010-0145-8. PubMed PMID: 20390359.

4. McGahern AM, Edwards CJ, Bower MA, Heffernan A, Park SD, Brophy PO, et al. Mitochondrial DNA sequence diversity in extant Irish horse populations and in ancient horses. Anim Genet. 2006;37(5):498-502. doi: 10.1111/j.1365-2052.2006.01506.x. PubMed PMID: 16978181.

5. Lei CZ, Su R, Bower MA, Edwards CJ, Wang XB, Weining S, et al. Multiple maternal origins of native modern and ancient horse populations in China. Anim Genet. 2009;40(6):933-44. doi: 10.1111/j.1365-2052.2009.01950.x. PubMed PMID: 19744143.

6. Jansen T, Forster P, Levine MA, Oelke H, Hurles M, Renfrew C, et al. Mitochondrial DNA and the origins of the domestic horse. Proc Natl Acad Sci U S A. 2002;99(16):10905-10. doi: 10.1073/pnas.152330099. PubMed PMID: 12130666; PubMed Central PMCID: PMC125071.

7. Kakoi H, Tozaki T, Gawahara H. Molecular analysis using mitochondrial DNA and microsatellites to infer the formation process of Japanese native horse populations. Biochem Genet. 2007;45(3-4):375-95. doi: 10.1007/s10528-007-9083-0. PubMed PMID: 17265183.

8. Khanshour AM, Cothran EG. Maternal phylogenetic relationships and genetic variation among Arabian horse populations using whole mitochondrial DNA D-loop sequencing. BMC Genet. 2013;14:83. doi: 10.1186/1471-2156-14-83. PubMed PMID: 24034565; PubMed Central PMCID: PMC3847362.

9. Prystupa JM, Hind P, Cothran EG, Plante Y. Maternal lineages in native Canadian equine populations and their relationship to the Nordic and Mountain and Moorland pony breeds. J Hered. 2012;103(3):380-90. doi: 10.1093/jhered/ess003. PubMed PMID: 22504109.

10. Achilli A, Olivieri A, Soares P, Lancioni H, Hooshiar Kashani B, Perego UA, et al. Mitochondrial genomes from modern horses reveal the major haplogroups that underwent domestication. Proc Natl Acad Sci U S A. 2012;109(7):2449-54. doi: 10.1073/pnas.1111637109. PubMed PMID: 22308342; PubMed Central PMCID: PMCPMC3289334.

11. Goto H, Ryder OA, Fisher AR, Schultz B, Kosakovsky Pond SL, Nekrutenko A, et al. A massively parallel sequencing approach uncovers ancient origins and high genetic variability of endangered Przewalski's horses. Genome Biol Evol. 2011;3:1096-106. doi: 10.1093/gbe/evr067. PubMed PMID: 21803766; PubMed Central PMCID: PMC3194890.

12. Liu G, Xu CQ, Cao Q, Zimmermann W, Songer M, Zhao SS, et al. Mitochondrial and pedigree analysis in Przewalski's horse population: implications for genetic management and reintroductions. Mitochondrial DNA. 2014;25(4):313-8.

13. Oakenfull EA, A. RO. Mitochondrial control region and 12S rRNA variation in Przewalski’s horse (Equus przewalskii). Anim Genet. 1998;29(6):456--9. doi: 10.1046/j.1365-2052.1998.296380.x.

14. Vila C, Leonard JA, Gotherstrom A, Marklund S, Sandberg K, Liden K, et al. Widespread origins of domestic horse lineages. Science. 2001;291(5503):474-7. doi: 10.1126/science.291.5503.474. PubMed PMID: 11161199.

15. Priskin K, Szabó K, Tömöry G, Bogácsi-Szabó E, Csányi B, Eördögh R, et al. Mitochondrial sequence variation in ancient horses from the Carpathian Basin and possible modern relatives. Genetica. 2010;138(2):211-8. doi: 10.1007/s10709-009-9411-x. PubMed PMID: 19789983.

16. Hill EW, Bradley DG, Al-Barody M, Ertugrul O, Splan RK, Zakharov I, et al. History and integrity of thoroughbred dam lines revealed in equine mtDNA variation. Anim Genet. 2002;33(4):287-94.

17. Alvarez I, Fernandez I, Lorenzo L, Payeras L, Cuervo M, Goyache F. Founder and present maternal diversity in two endangered Spanish horse breeds assessed via pedigree and mitochondrial DNA information. J Anim Breed Genet. 2012;129(4):271-9. doi: 10.1111/j.1439-0388.2012.00995.x. PubMed PMID: 22775259.

18. Devi KM, Ghosh SK. Molecular phylogeny of Indian horse breeds with special reference to Manipuri pony based on mitochondrial D-loop. Mol Biol Rep. 2013;40(10):5861-7. doi: 10.1007/s11033-013-2692-2. PubMed PMID: 24068432.

19. Bowling AT, Del Valle A, Bowling M. A pedigree-based study of mitochondrial D-loop DNA sequence variation among Arabian horses. Anim Genet. 2000;31(1):1-7.

20. Mirol PM, Peral García P, Vega-Pla JL, Dulout FN. Phylogenetic relationships of Argentinean Creole horses and other South American and Spanish breeds inferred from mitochondrial DNA sequences. Anim Genet. 2002;33(5):356-63. PubMed PMID: 12354144.

21. Kavar T, Habe F, Brem G, Dovč P. Mitochondrial D-loop sequence variation among the 16 maternal lines of the Lipizzan horse breed. Animal Genetics. 1999;30(6):423-30. doi: 10.1046/j.1365-2052.1999.00557.x.

22. Kavar T, Brem G, Habe F, Solkner J, Dovc P. History of Lipizzan horse maternal lines as revealed by mtDNA analysis. Genet Sel Evol. 2002;34(5):635-48. doi: 10.1051/gse:2002028. PubMed PMID: 12427390; PubMed Central PMCID: PMC2705438.

23. Bower MA, Campana MG, Whitten M, Edwards CJ, Jones H, Barrett E, et al. The cosmopolitan maternal heritage of the Thoroughbred racehorse breed shows a significant contribution from British and Irish native mares. Biol Lett. 2011;7(2):316-20. doi: 10.1098/rsbl.2010.0800. PubMed PMID: 20926431; PubMed Central PMCID: PMC3061175.

24. Campana MG, Stock F, Barrett E, Benecke N, Barker GW, Seetah K, et al. Genetic stability in the Icelandic horse breed. Anim Genet. 2012;43(4):447-9. doi: 10.1111/j.1365-2052.2011.02266.x. PubMed PMID: 22497715.

25. Cozzi MC, Strillacci MG, Valiati P, Bighignoli B, Cancedda M, Zanotti M. Mitochondrial D-loop sequence variation among Italian horse breeds. Genet Sel Evol. 2004;36(6):663-72. doi: 10.1051/gse:2004023. PubMed PMID: 15496286; PubMed Central PMCID: PMC2697199.

26. Ishida N, Hasegawa T, Takeda K, Sakagami M, Onishi A, Inumaru S, et al. Polymorphic sequence in the D-loop region of equine mitochondrial DNA. Anim Genet. 1994;25(4):215-21.

27. Bigi D, Perrotta G, Zambonelli P. Genetic analysis of seven Italian horse breeds based on mitochondrial DNA D-loop variation. Anim Genet. 2014;45(4):593-5. doi: 10.1111/age.12156. PubMed PMID: 24702170.

28. Perez-Gutierrez LM, De la Pena A, Arana P. Genetic analysis of the Hispano-Breton heavy horse. Anim Genet. 2008;39(5):506-14. doi: 10.1111/j.1365-2052.2008.01762.x. PubMed PMID: 18680492.

29. Georgescu SE, Manea MA, Dudu A, Costache M. Phylogenetic relationships of the Hucul horse from Romania inferred from mitochondrial D-loop variation. Genet Mol Res. 2011;10(4):4104-13. doi: 10.4238/2011.October.31.7. PubMed PMID: 22057995.

30. Glazewska I, Wysocka A, Gralak B, Sell J. A new view on dam lines in Polish Arabian horses based on mtDNA analysis. Genet Sel Evol. 2007;39(5):609-19. doi: 10.1051/gse:2007025. PubMed PMID: 17897600.

31. Luís C, Bastos-Silveira C, Cothran EG, Oom MoM. Iberian origins of New World horse breeds. J Hered. 2006;97(2):107-13. doi: 10.1093/jhered/esj020. PubMed PMID: 16489143.

32. Royo LJ, Alvarez I, Beja-Pereira A, Molina A, Fernandez I, Jordana J, et al. The origins of Iberian horses assessed via mitochondrial DNA. J Hered. 2005;96(6):663-9. doi: 10.1093/jhered/esi116. PubMed PMID: 16251517.

33. Morelli L, Useli A, Sanna D, Barbato M, Contu D, Pala M, et al. Mitochondrial DNA lineages of Italian Giara and Sarcidano horses. Genet Mol Res. 2014;13(4):8241-57. doi: 10.4238/2014.October.20.1. PubMed PMID: 25366719.

34. Lopes MS, Mendonca D, Cymbron T, Valera M, da Costa-Ferreira J, Machado Ada C. The Lusitano horse maternal lineage based on mitochondrial D-loop sequence variation. Anim Genet. 2005;36(3):196-202. doi: 10.1111/j.1365-2052.2005.01279.x. PubMed PMID: 15932397.

35. Criscione A, Moltisanti V, Chies L, Marletta D, Bordonaro S. A genetic analysis of the Italian Salernitano horse. Animal. 2015;9(10):1610-6.

36. Luís C, Bastos-Silveira C, Costa-Ferreira J, Cothran EG, Oom MM. A lost Sorraia maternal lineage found in the Lusitano horse breed. J Anim Breed Genet. 2006;123(6):399-402. doi: 10.1111/j.1439-0388.2006.00612.x. PubMed PMID: 17177696.
