# Supplementary material for: An Overview of Ten Italian Horse Breeds through Mitochondrial DNA
Source: PLoS One. 2016 Apr 7;11(4):e0153004. doi: 10.1371/journal.pone.0153004 (PMC4824442; doi:10.1371/journal.pone.0153004)
Supplement: S5 Table — (DOCX) [file pone.0153004.s006.docx]

**S5 Table. A geographic comparison of haplogroup frequencies (%)^a^.**

| Macroarea | Italian Peninsula | Southwestern Asia | Continental Europe |
| --- | --- | --- | --- |
| A | 6.95 | 5.56 | 5.83 |
| B | 9.27 | 4.86 | 6.67 |
| C | 3.09 | 6.25 | 1.11 |
| D | 1.54 | 1.39 | 8.61 |
| E | 2.70 | 3.47 | 0.28 |
| G | 12.74 | 7.99 | 5.28 |
| H’I | 4.63 | 10.76 | 9.72 |
| J’K | 2.70 | 5.21 | 0.00 |
| L | 24.71 | 18.06 | 31.11 |
| M | 8.49 | 3.82 | 8.33 |
| N | 4.25 | 2.43 | 8.89 |
| O’P | 8.11 | 6.94 | 1.11 |
| Q | 9.27 | 10.42 | 8.61 |
| R | 1.16 | 2.08 | 0.56 |
| Unclassified | 0.39 | 10.76 | 3.89 |
| Total Samples | 259 | 288 | 360 |

^a^ Source data from S3 and S4 Tables.
